# An optimized design for motivated broadband LPDA antenna

**DOI:** 10.1038/s41598-024-57449-5

**Published:** 2024-03-28

**Authors:** Islam M. Ibrahim, Mohamed I. Ahmed, Hala M. Abdelkader, M. M. Elsherbini

**Affiliations:** 1 Department of Electrical Engineering, Shoubra Faculty of Engineering, Benha University, Cairo, 11629 Egypt; 2 Department of Electrical Engineering, The Egyptian Academy for Engineering and Advanced Technology (EAEAT), Cairo, 3056, Egypt; 3 Microstrip Department, Electronics Research Institute, El-Nuzha, Cairo, 11843 Egypt; 4 Electronics and Communication Program, Faculty of Engineering, Egypt University of informatics, New Administrative Capital, Knowledge City, Egypt

**Keywords:** Broadband, Millimeter wave, V-Band applications, Director Units, LPDA, Engineering, Electrical and electronic engineering

## Abstract

This paper presents a super wideband and high-gain log periodic dipole array (LPDA) antenna. The overall structure of the antenna was constructed using microwave studio computer simulation technology. The optimal sizes of the planned antenna are 39 × 10× 0.254 mm^3^. The engineered antenna arrangement is implemented on an RT5880 substrate as a dielectric medium. The LPDA is arranged in four arms that are equally spaced on both lines. The main 50Ω feeder line is partially grounded at the back of the substrate. A combination of circular director units is being studied and tuned in a regular pattern at a predefined distance from the antenna. An improvement in gain of 3 dBi is the response of the director units. The Conformist LPDA is adjusted to achieve a wide range of millimeter wave bands ranging from 40 to over 70 GHz. The antenna resonates at 60 GHz, where the maximum realized gain of 14.97 dBi is attained. The antenna was tested for utilization in the V-band involving wireless personal area network (WPAN) applications recommended by IEEE 802.11ad and IEEE 802.15.3c. The outcomes of the constructed antenna elements' tests and simulations agree fairly well. The proposed layout works better than previous efforts in this field.

## Introduction

Currently, wireless communication systems are witnessing great development in all fields and will continue to advance, and their wide range of utilization is nearly unfathomable. A wide-band, compact, and dual-polarized log-periodic dipole array antenna is designed to cover the spectrum from 0.99 GHz to 12 GHz. The impedance bandwidth of the antenna is 169.5%. The antenna transverse dimension is lowered to 80%^1^. A log-periodic dipole array antenna is designed and fabricated on the textiles to cover the frequency band from 0.9–3 GHz. The antenna has an average gain of 5.9 dBi and a 3.33:1 impedance bandwidth^2^. A wideband and high-efficiency polarization conversion metasurface is proposed. The metasurface has a polarization conversion ratio greater than 90% between 7.4 GHz and 17 GHz^3^. A log-periodic Koch dipole array is designed with a compact dimensions. The overall dimension of the single dipole component is minimized with the new Koch split folding dipole and the T-shaped top structure. The radio frequency range in which the antenna functions is 195–848 MHz^4^. A planar log-periodic slot antenna is implemented to be used for UWB (ultra-wideband) purposes. In low frequencies, the resonant frequencies can be reduced by around 12.3% when compared to a standard log-periodic slot antenna featuring regular-shaped edges on the slots^5^. A 4 × 4 array loaded with near-zero-index metamaterial enhances and increases the gain. The design is built on FR-4 material. The result indicates that this antenna has a peak gain of 14 dBi at the operating frequency of 5.8 GHz^6^. The patch is designed on an FR-4 substrate. A ground plane and a patch of metal foil make up the wide-band antenna. The design has a spectrum in the range of 4–10 GHz. The antenna is used for WLAN applications^7^. The antenna is designed to operate at an operational frequency of 60 GHz. A dielectric lens is loaded into the LPDA antenna to improve the gain. The antenna is fabricated for V-band applications^8^. 4 × 4 LPDA array antennas are implemented and have a peak gain of 15.5 dBi. The antenna has an impedance bandwidth of 32.5% that covers a frequency range of 32.5–45 GHz^9^. The antenna is modeled with dimensions of 50 × 50 mm^2^ and a 0.8 mm thickness, built on FR-4 substrate as a dielectric medium. A balun circuit is designed to facilitate impedance matching. The total realized gain is 6.5 dBi^10^. 1 × 4 antenna array designed for Ka-band applications^11^. The configuration for the LPDA antenna is established in the frequency band of 0.7–8 GHz. The peak realized gain of the LPDA has reached 5.5 dBi^12^. The three-element LPDA antenna is designed on a substrate of 80 × 80 × 1.6 mm^3^ as a dielectric. The ground plane is 0.01 mm thick and has the same length and width as the substrate. The antenna has a frequency range of 12–18 GHz^13^. A broad-band and efficient land-storfer-printed LPDA antenna is planned and furnished with zero-index metamaterial. The antenna has a peak gain of 2.2 dBi and a frequency range of 26.5–40 GHz. The antenna is used for cellular system applications^14^. The LPDA antenna is designed and fully equipped with non-resonant metamaterial (MTM) inclusions. Metamaterials improve the gain by at least 1.6 dBi up to 4 dBi at 27 GHz^15^. The antenna is printed on RT5880 with a relative permittivity of 2.2 and a 0.003 mm thickness. A narrow-band patch antenna is designed to operate at 60 GHz. The antenna has a peak gain of 8.82 dBi. The antenna is used for 5G wireless applications^16^. The LPDA antenna is imprinted on the FR-4 substrate as a dielectric. It has a significant realized gain of 8.42 dBi. The antenna is applicable for IOT devices^17^. The antenna has dimensions of 10 × 10 × 0.245 mm^3^. The antenna's operational frequency falls between 33 and 43 GHz^18^. An 11-element log-periodic dipole array with dimensions of 90 × 52 mm^2^ is implemented on a Kapton substrate and operates between the frequencies of 2.75–3.53 GHz and 4.6–6.2 GHz. The antenna has an actual gain of 6 dBi and an end-fire radiation pattern^19^. The single-element and two-port MIMO antenna is fabricated on RO3003TM with a thickness of 0.25 mm. The antenna operates in the bands around 28 and 38 GHz^20^. The Truncated Log-Periodic Dipole Array Antenna is modeled to operate in a frequency band of 760 MHz to 18 GHz^21^. A MIMO antenna is fabricated on Roger's 4350B with a size of 11.4 × 5.3 mm^2^. The antenna operates at 29 GHz and is utilized for 5G communication^22^. A broadband LPDA is tested to operate in the range of 500 MHz to 20 GHz. The operating frequency range is 4–20 GHz, and its realized gain is 6.1 dBi^23^. A widespread antenna with a substantial gain of 10.7 dBi is designed for 5G systems. The antenna impedance matching is 35.53%. The design covers a wide band from 23.4 to 13.92 GHz. The antenna's operational frequency is 28 GHz^24^. The antenna is implemented on a Rogers RT 5870 with an optimal thickness of 2.33 mm. It has resonant frequencies of 28 GHz, 38 GHz, and 55 GHz, which are allotted for 5G and V-band communications^25^. A Yagi antenna was released for 28/38 GHz. The element is a perforated strip dipole with an extra strip that is capacitive end-coupled. The antenna consists of one director and two reflectors in a configuration of triangles^26^. The LPDA antenna was designed for 5G applications. The antenna design has a bandwidth of 21–37 GHz. The antenna has a maximum gain of 12–14.5 dBi^27^. A broad-range LPDA antenna is imprinted on the RO5880 dielectric. The antenna has five arms loaded with metamaterials that radiate from 26 to 39 GHz. The. The MIMO LPDA was implemented to enhance the realized gain. The peak gain of MIMO has reached 11 dBi^28^. A broadband MIMO antenna was implemented for 5G communication utilizing Meta Surface. A 2 × 2 non-uniform meta-surface is positioned at the rear of the MIMO arrangement to enhance its radiation characteristics. The spectrum of the antenna spans from 23.5 to 29.4 GHz^29^. A multiband antenna that operates at the central frequencies of 34 GHz, 62 GHz, 76 GHz, and 93.7 GHz. It shows a discernible gain of 7.48 dBi, 6.81 dBi, 8.88 dBi, and 10.90 dBi^30^. The LPDA prototype is fabricated to cover the range of 24–39 GHz. The impedance matching of the antenna reached 49.6%. The total gain for the antenna rose to 13 dBi^31^. A T-shaped antenna is designed with a small dimension of 18.5 × 24 mm^2^ and a dielectric constant of 2.3 on a thin Rogers's 5880 substrate. The antenna has a peak realized gain of 11.5 dBi^32^. The antenna arrangement has a size of 28.3 × 28.3 × 0.508 mm^3^. The antenna has a nominal bandwidth of 3.9 GHz. Its spectrum extended from 26.5 GHz to 30.4 GHz. At 28 GHz, the antenna achieved a maximum gain of 11 dBi and is used for 5G communications^33^. The design of a multiband mm-wave antenna involves spanning bands, namely 24.34–29 GHz, 33–40 GHz, and Ku-bands (14.44–20.98 GHz), which are potential spectrum bands for 5G wireless communications^34^. A wideband dual-reflector antenna is designed to operate in the mm-wave band throughout 28 and 39 GHz and is used for Ka-band applications. The antenna functions with a consistent gain^35^. DRs antennas are constructed on a Taconic RF-35 substrate using a flipped trapezoidal patch. The antenna spectrum ranges from 4.85 GHz to 10.88 GHz. The antenna's operational frequency is between 6.14 GHz and 10.52 GHz. The prototype exhibits an overall gain of 8 dBi at 10.2 GHz^36^. The MIMO antenna array is constructed to operate in the 37 GHz frequency spectrum to be applicable for 5G communication uses. The peak gain for the design has reached 12.8 dBi by using an array arrangement with four components^37^. The initial and final frequencies of the surface currents in the two bands, which are 1.7 GHz, 4 GHz, 5.5 GHz, and 6.5 GHz, respectively, show that the greatest currents are evidently flowing near the antenna. The strength of the currents in the central regions decreases further as frequency rises. One method of reducing the size of antennas is to curve the current paths. A second ring is attached to the inner side of the first ring to lengthen the electrical route length of the current and so raise the inductance^38^. The antenna is designed for the operating band, which spans 1.65 GHz to 160 GHz and has a thinner height. The antenna's ratio bandwidth ranges from 96.96:1 to 115.10:1, covering a vast frequency range^39^. The planar Vivaldi antenna is established to span the range between 55 and 84 GHz. The antenna has a significant gain of 7 dBi with a radiation efficiency of over 80%^40^. The antenna is constructed with a variety of flared-out feed lines and an unconventional circular form. The etched-out rings with defined width are precisely incorporated into the patch to regulate the antenna's resonance properties^41^. The antenna is designed with dimensions of 26 × 50 mm^2^ to cover the range from 2.23 GHz to more than 100 GHz. The antenna is utilized to create a MIMO antenna with no decoupling structures and just two cat-shaped geometries^42^. A log-periodic dipole array is printed on RT 5880 and used for the Ku/K band. The antenna's entire substrate measurements are 55 × 45 mm^2^. The antenna has an impedance bandwidth of 1.3% (12.82–12.98 GHz), 3.1% (13.54–13.96 GHz), 2.3% (14.81–15.15 GHz), 4.5% (17.7–18.52 GHz), and 4.6% (21.1–22.1 GHz)^43^. A broad-band log periodic antenna is implemented to be applicable for 5G femtocells. The model has a fractional band width of 34.73%. The frequency ranges in which the antenna operates are (26, 27.5, 29, 30, 32, 33, 35, and 38.1GHz)^44^. A small, wide-bandwidth 5G planar quad element (MIMO) antenna is designed in the form of a tree. Four distinct arcs are used in the layout to provide a wide bandwidth. The antenna's bandwidth spans from 23 to 40 GHz. The overall gain was 10.58 dBi, 8.87 dBi, and 11.45 dBi at 28 GHz, 33 GHz, and 38 GHz^45^. The antenna layout is configured to operate at a wide range of wireless uses, including WiMAX (3.3–3.6 GHz), 5G (3.3–3.7 GHz), WLAN (5.15–5.825 GHz), UWB (3.1–10.6 GHz), Ku–(12–18 GHz), K–(18–27 GHz), Ka–(27–40 GHz), V–(40–75 GHz), and W–(75–110 GHz). The antenna improved a gain between 3.22 and 7.23 dBi^46^.

The paper is organized as follows: Section "Antenna configurations and design for unloaded LPDA". Explain the LPDA antenna design without director units. Section "The proposed LPDA antenna with loaded straight circular director" shows the LPDA design improved by loading small-sized circular director units. Section "Parametric study" displays the parametric study for the LPDA design to get the antenna response for the realized gain, S-parameter, and radiation pattern. Section "Results and discussions"; Presents the simulation's and measurement results and a comparison of the various implementations from the literature with the current work. Finally, Section "Conclusion" displays the work conclusions.

The novelty of the paper is that the LPDA antenna was implemented on a thin RT5880 substrate with small losses and a small dielectric thickness of 0.254 mm. The LPDA improves the realized gain and radiation pattern. The prototype is used for V-band involving wireless personal area network (WPAN) applications recommended by IEEE 802.11ad and IEEE 802.15.3c. The antenna covers a super-wide band range from 40 to 70 GHz. A regular array of small circular director units is loaded on the LPDA, which has great effects on enhancing and increasing the realized gain. The gain is improved by using director units at roughly 15 dBi. The antenna has a large amount of bandwidth and broadband coverage, which makes it have greater efficiency as well. The antenna dimensions are compact. The antenna design cost is minimal compared to dielectric lenses. The proposed work offers satisfactory results over previous ones.

## Antenna configurations and design for unloaded LPDA

**Figure 1:**
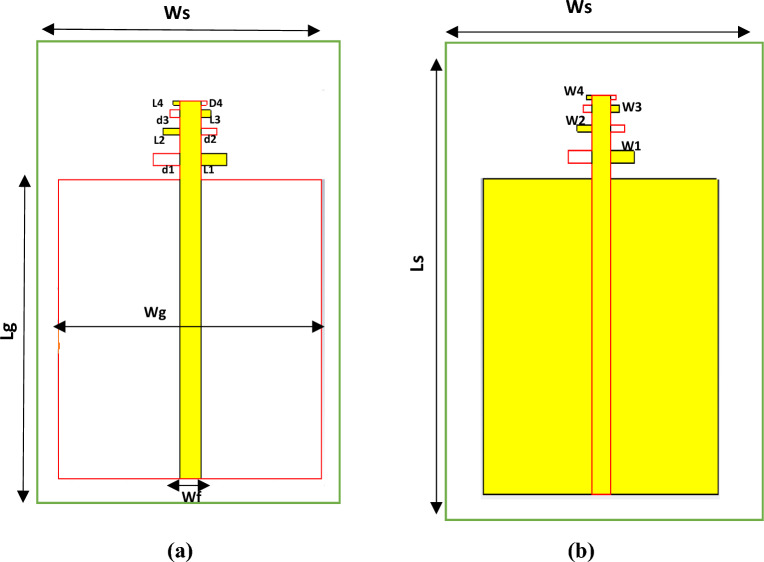
(**a**) LPDA configuration without Directors, (**a**) Front View, (**b**) Back view.

The main objective of the proposed LPDA antenna is to accommodate communication requirements that operate in a frequency range of 40–70 GHz. The entire LPDA antenna assembly is embedded with a minimal loss material (RT5880) with a size of 39 × 10 × 0.254 mm^3^, as shown in Fig. 1. Its thickness is 0.254 mm, its metal thickness is 0.035 mm, its dielectric constant is 2.2, and its loss tangent is 0.0009. All antenna dimensions are listed in the Table 1. The engineered antenna is then based on the modelling of four arms with a main feeding line of 50 Ω. A Partial ground is at the back of the substrate, intended to accomplish super-wide band characteristics. In summary, partial grounds release a portion of the substrate energy. The lower energy storage in the materials causes a drop in the quality factor (Q). As the Q factor decreases, the bandwidth increases. The dipole elements (N) (N = 4) and the spacing factor (r). The largest LPDA length, L1, is calculated from the lowest resonant frequency f_min_ and the spacing factor r, which determines how far apart the dipole components are from one another. Figure 1 depicts the front and back views of a real LPDA design with four arms without director units. The variables apply to the nth dipole component of the planned LPDA antenna: Ln, Wn, and Sn. The acronym Wf refers to the micro-strip feeder width. The 50-Ω feeding line was modified in order to correlate with the measurements from the vector network analyzer to achieve the matching. The corresponding ratio of the widths and lengths of the two adjacent dipole parts is the geometric constant, whereas the distinction parameter is the distance between each of the relatively dipole elements. On each side of the substrate's dipole arm, it is evenly positioned along the parallel microstrip transmission lines. The scaling factor for LPDA can be calculated from the equation^47^.

**Table 1:** Dimensions for unloaded LPDA.

Parameters	Values (mm)
W_S_	10
L_s_	39
W_f_	0.78
Wg	10
Lg	10.21
L1	1
L2	0.60
L3	0.371
L4	0.226
W1	0.39
W2	0.242
W3	0.242
W4	0.12
d1	0.49
d2	0.61
d3	0.376
d4	0.171
H	0.254

1 $$\tau =\frac{{L}_{2}}{{L}_{1}}=\frac{{L}_{n+1}}{{L}_{n}}=\frac{{S}_{n+1}}{{S}_{n}}=\frac{{f}_{n}}{{f}_{n+1}}$$

The spacing factor can be calculated as:

2 $$\sigma =\frac{{S}_{1}}{2{L}_{1}}=\frac{{S}_{n}}{2{L}_{n}}=\frac{{S}_{n+1}}{{S}_{n}}$$

The apex angle of the LPDA:

3 $$\mathrm{\alpha }={tan}^{-1}\frac{{L}_{n}-{L}_{n+1}}{2{S}_{n}}={tan}^{-1}\frac{{L}_{n}(1n\tau )}{2{S}_{n}}={tan}^{-1}\frac{1t\tau }{4\sigma }$$

The total number of elements of the antenna design:

4 $${\text{N}}=(\frac{Log\left(\frac{{f}_{1}}{{f}_{2}}\right)}{Log\left(\tau \right)}+1$$

The spacing between elements n:

5 $${S}_{n}=2{L}_{n}\sigma$$

The length of elements:

6 $${L}_{N}=\uptau {L}_{N-1}=\frac{C}{{f}_{max}}$$

7 $${\text{G}}=10{log}_{10}\left(4\pi \frac{{U}_{max}}{{P}_{rad}}\right)$$

The gain equation:

8 $${\text{U}}=\frac{1}{2} \frac{{E}^{2}{r}^{2}}{{n}_{o}}$$

The radiation intensity, where ${n}_{o}$ is the intrinsic impedance, G is the realized gain and ${P}_{rad}$ is the power radiated.

## The proposed LPDA antenna with loaded straight circular director

**Figure 2:**
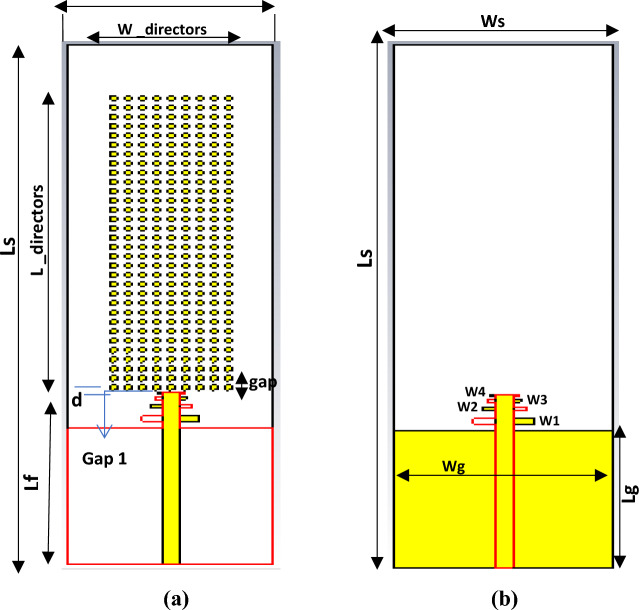
Loaded LPDA Array Configuration Model, (**a**) Front View, (**b**) Back View.

The engineered recommended LPDA antenna is an optimized design that is modified and loaded with director units. A regular series of circular director units with optimized and very small dimensions is summarized in Table 2. The director units are packed with the LPDA. The directors are positioned at the front of the antenna at a predefined and tuned distance that is systematically separated from the antenna. It is preferable to focus the surface current through the series of directors, which increases gain, decreases the back lobe, and obtains a stable radiation pattern. A great trial and parametric study for the directors to achieve the best optimum value for the gain. The study starts with one director, and then a row of director units is formed with an investigational relocation between every director and every other row. The director arrangement avalanches and reaches fifteen simultaneous straight rows, as depicted in Fig. 2, guaranteeing high efficiency, impedance matching, and improving the substantial gain for the antenna. Directors reflect the radio waves with an alternate phase, changing the electromagnetic radiation patterns of the waves. The outcome is positive interference, which increases gain by strengthening the overall signal. By creating a director's consequences, the director was created to supplement the dielectric lenses and metamaterials with the objective of obtaining the greatest gain. The spacing between the director units is equally and symmetrically spaced to improve performance and decrease losses.

**Table 2:** Dimensions for loaded LPDA.

Parameters	Values (mm)
L_dir	22.10
W_dir	6
gap_dir	0.3
gap1_dir	0.725268
r_dir	0.2
d_dir	0.4

## Parametric study

**Figure 3:**
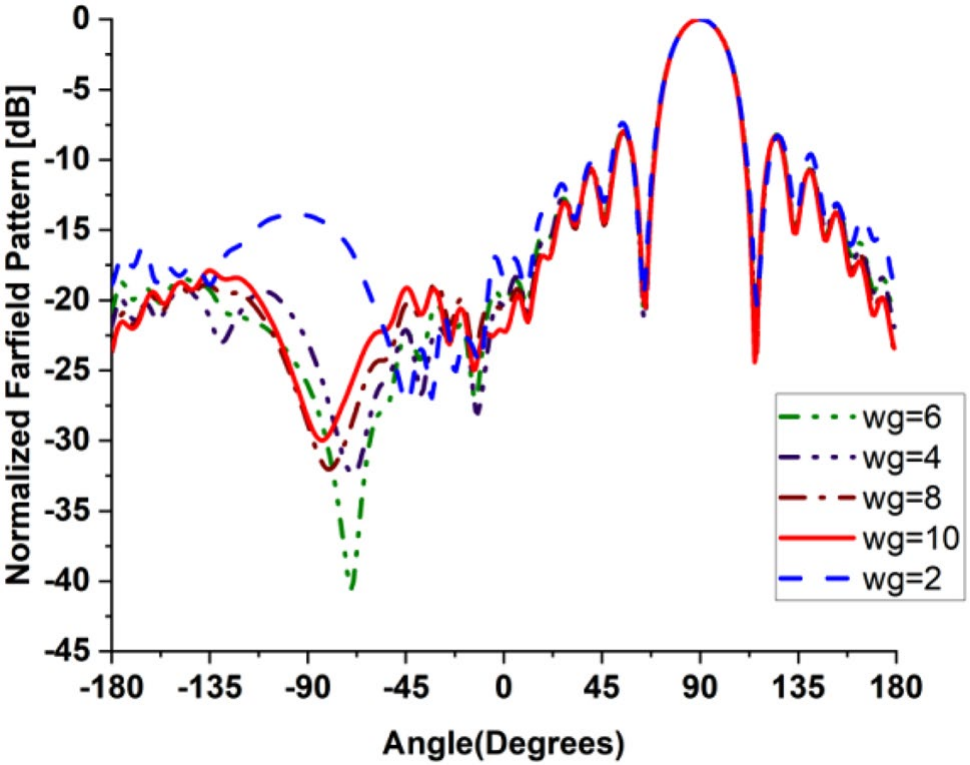
2D Simulated E-Plane Radiation pattern of wg study for loaded LPDA at (60GHz).

**Figure 4:**
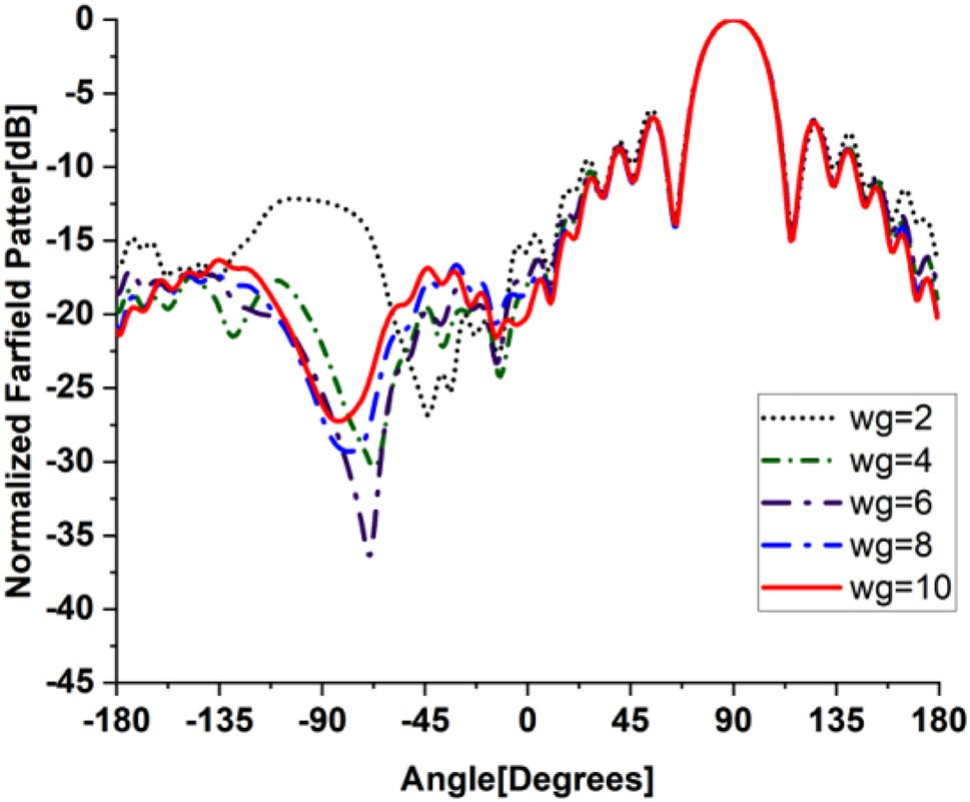
2D Simulated E-Plane Radiation pattern of wg for unloaded LPDA at (60GHz).

**Figure 5:**
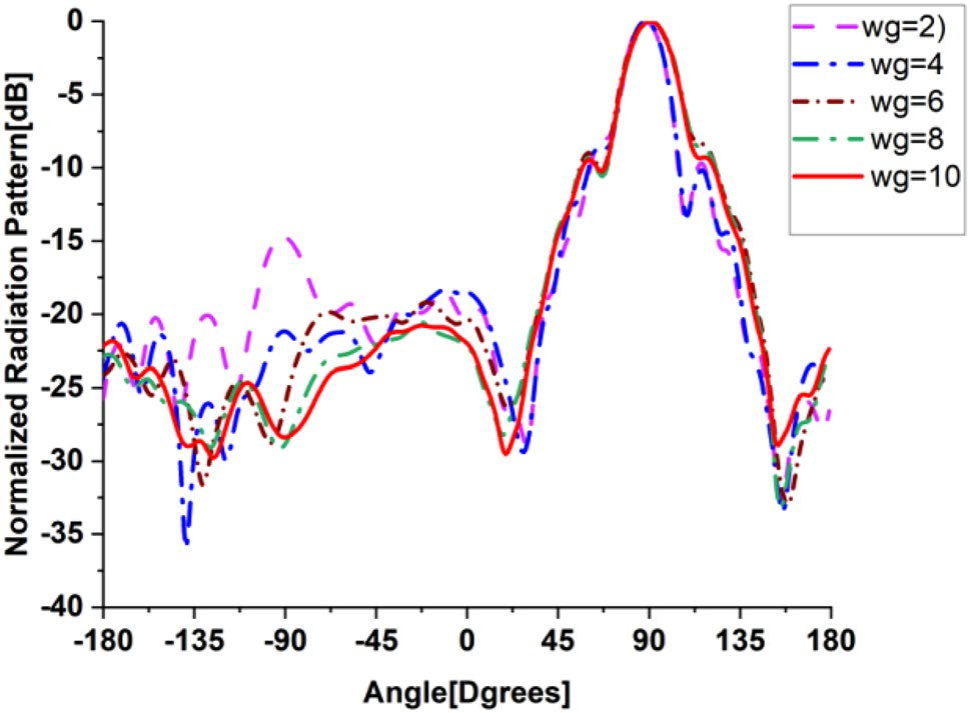
2D H-Plane Simulated Radiation pattern with different wg for loaded LPDA at (60GHz).

**Figure 6:**
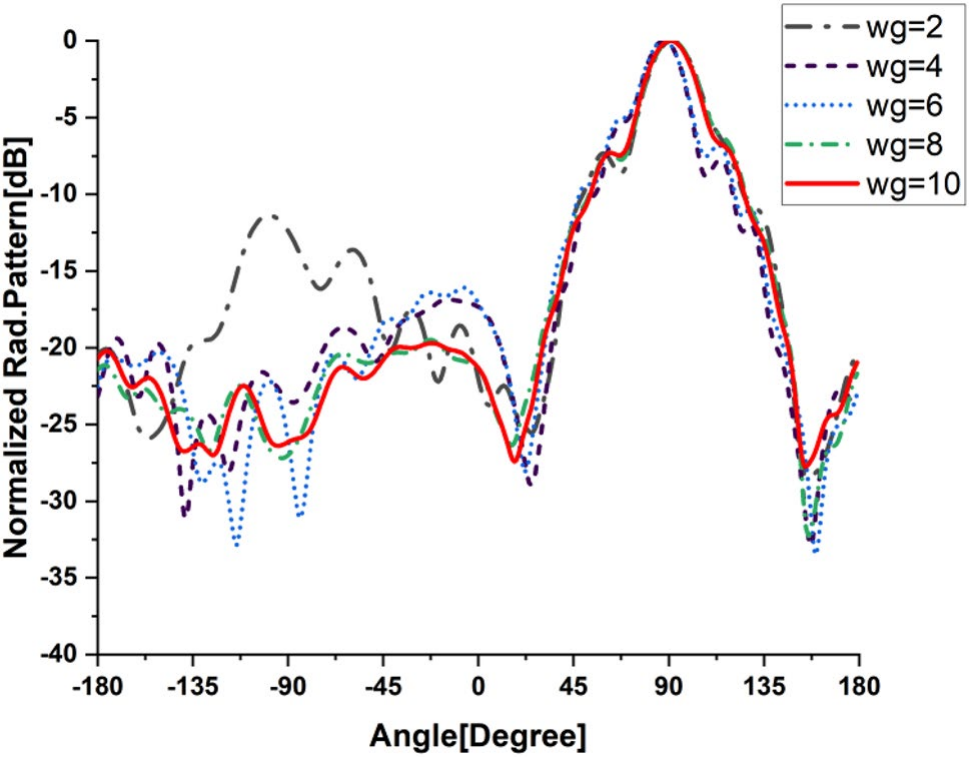
2D H-Plane Simulated Radiation pattern with different wg for unloaded LPDA at (60GHz).

**Figure 7:**
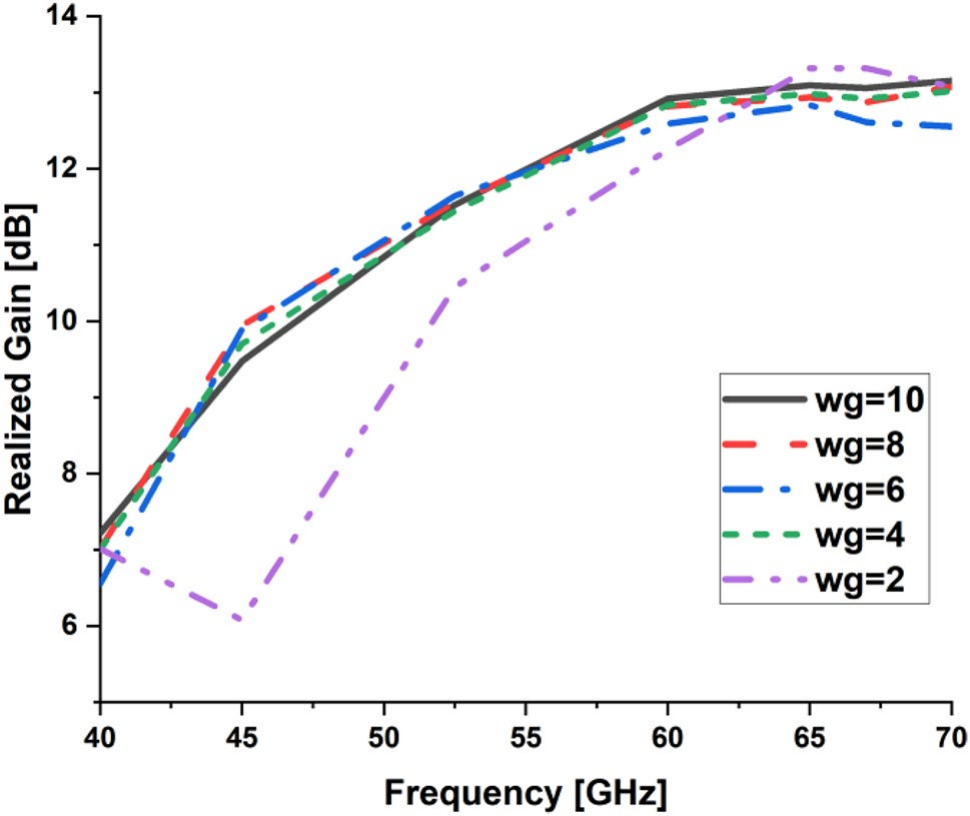
Simulated Realized gain for unloaded LPDA with different Wg values.

**Figure 8:**
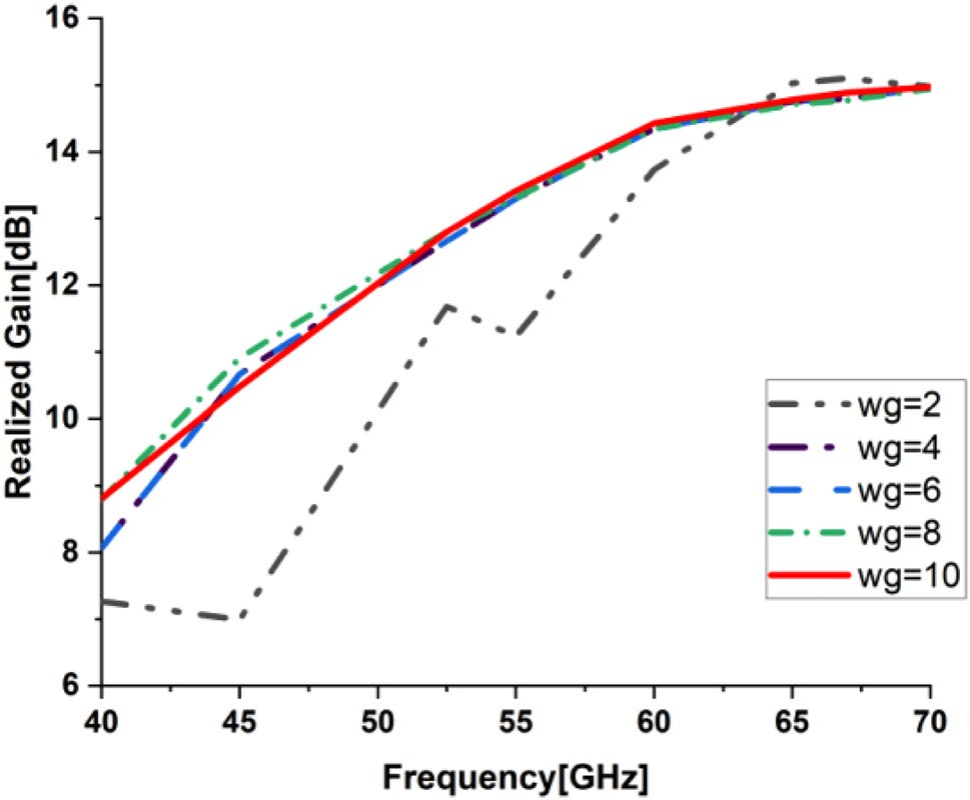
Simulated Realized gain for loaded LPDA with different Wg values.

**Figure 9:**
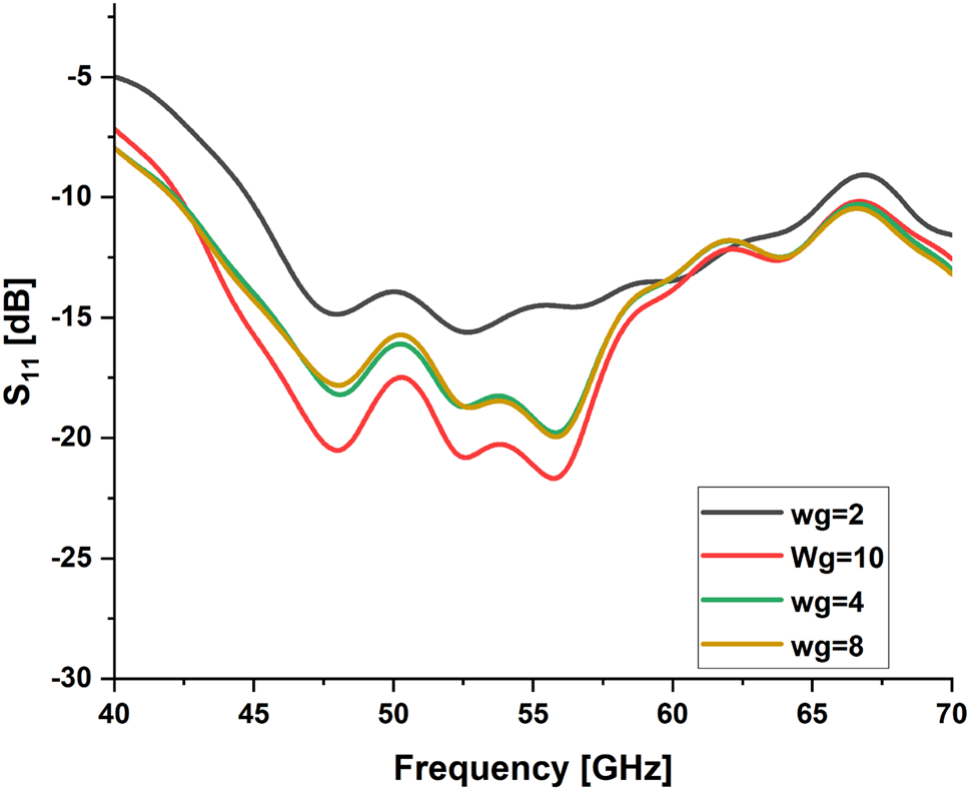
Simulated S_11_ for loaded LPDA with different Wg values.

In the present work, our primary goal is to utilize the performance of the antenna. A great parametric study was executed to improve the performance of the engineered design. The study has been done on loaded and unloaded LPDA. The unloaded LPDA antenna has a realized gain of 12.78 dBi. The director units are microstrip patches forming a circle with a 0.2 mm radius. All director units are subtly integrated into the antenna array and thus proportionately placed, as seen in Fig. 2. The realized gain obtained from the antenna relatively marginally increases whenever the director units are duplicated. A single set of directors is placed close to the antenna at an optimum tuned spacing of 0.182568 mm between director units and the LPDA. There were 9 director units in each row. The number of director units was raised one row at a time. As a result, we inserted an additional row and discovered an additional rise in gain. The directors studied till the rows reached 32. As a result, the loaded LPDA antenna's total realized gain reaches 14.97 dBi. The parametric study shows a stable normalized radiation pattern. Our design is based on decreasing and diminishing the back lobe, and the authors think that the worst level of the side lobe, or minor lobe, is directly behind the main lobe and in the opposite direction from the main lobe. Furthermore, the design contains a partial ground, which reduces the back lobe of the antenna as it suppresses surface wave diffraction from the edges of the antenna ground plane. Study the "Wg" starts with 2, 4, 6, 8, and 10 mm. It is noticed that the best optimum dimension is at Wg = 10 mm as it decreases the back lobe (minor) radiation of the antenna, as shown in Figs. 3, 4, 5, And 6 for the E-plane and the H-plane. The design gives great matching bandwidth and a high gain after five parametric studies for Wg with loaded and unloaded LPDA, as seen in Figs. 7 and 8. The realized gain for unloaded LPDA shown in Fig. 7 is enhanced by the study of the Wg. The realized gain for an unloaded antenna reaches 12.78 dBi at 60 GHz for the value of Wg = 10, as shown in Fig. 7. When the LPDA model is modified by adding circular director units on the Y-axis, the gain is enhanced to 14.97 dBi, as shown in Fig. 8. Also, the wg parameter greatly affects matching, where we obtain the best value for S_11_ below − 10 dB with a parametric sweep for wg, as seen in Fig. 9.

## Results and discussions

**Figure 10:**
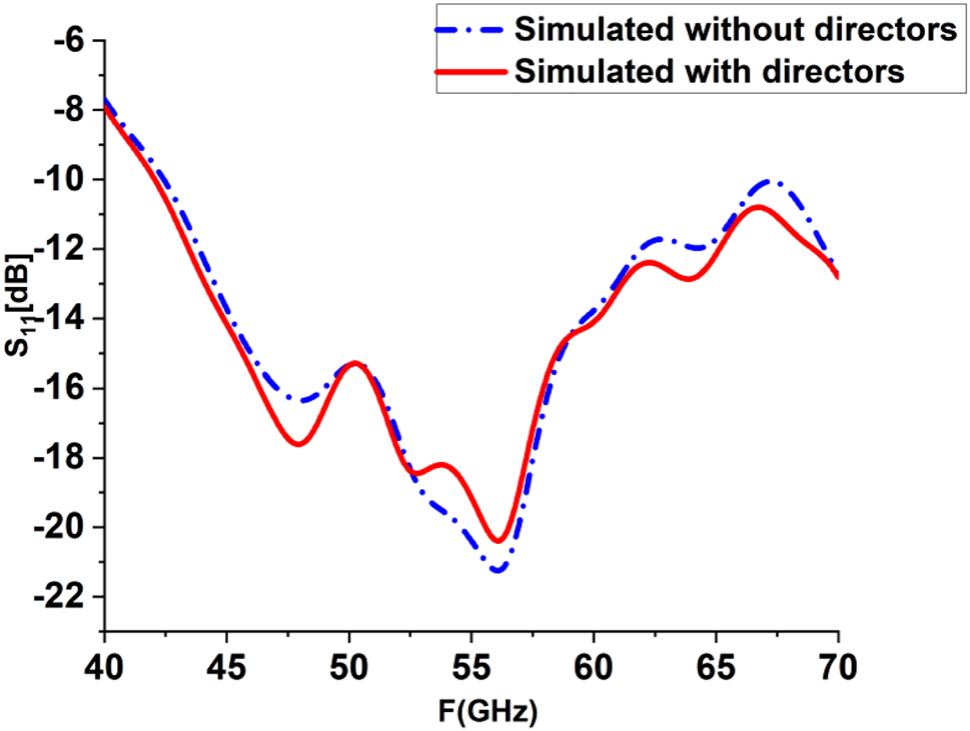
Simulated S-Parameters for loaded and unloaded LPDA.

A realistic summary of the planned antenna performance is attained through perceptive analysis, alluring current distribution characteristics, and various frequency resonances. The next step was to fabricate the antenna in order to verify the physical performance and confirm the previously indicated simulation results. From the measured and simulated results, it's obvious that the antenna spectrum ranges from 40 to 70 GHz, and S_11_ is below − 10 dB. It is noticed that the S_11_ is roughly − 21.2 dB without a loaded director; the value of the S_11_ was obtained at around − 20 dB for loaded LPDA, as shown in Fig. 10. The S_11_ doesn't change a lot with or without director cells. It is obvious that the changes in S-parameters are slight. Wg is one of the most important design parameters as it has a big impact on the antenna's performance; including gain, back lobe, and frequency band. After a thorough investigation of this parameter, it was found that the best optimum dimension is at Wg = 10 mm, as it decreases the back lobe radiation of the antenna.

**Figure 11:**
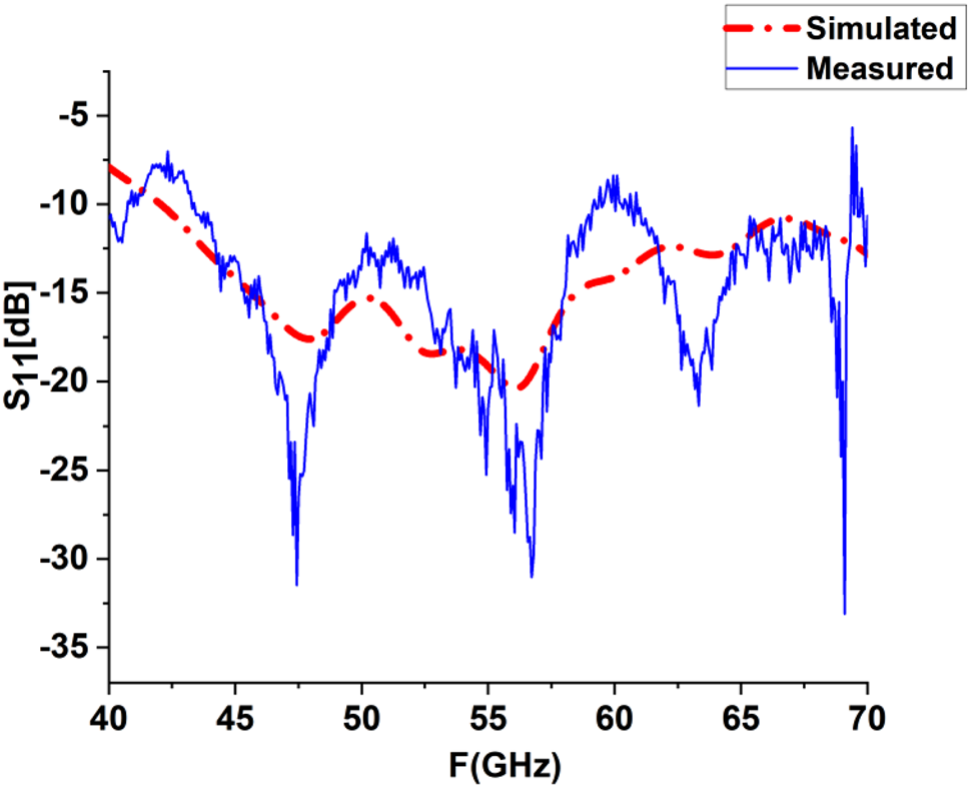
Simulated &Measured S-Parameters for loaded LPDA.

**Figure 12:**
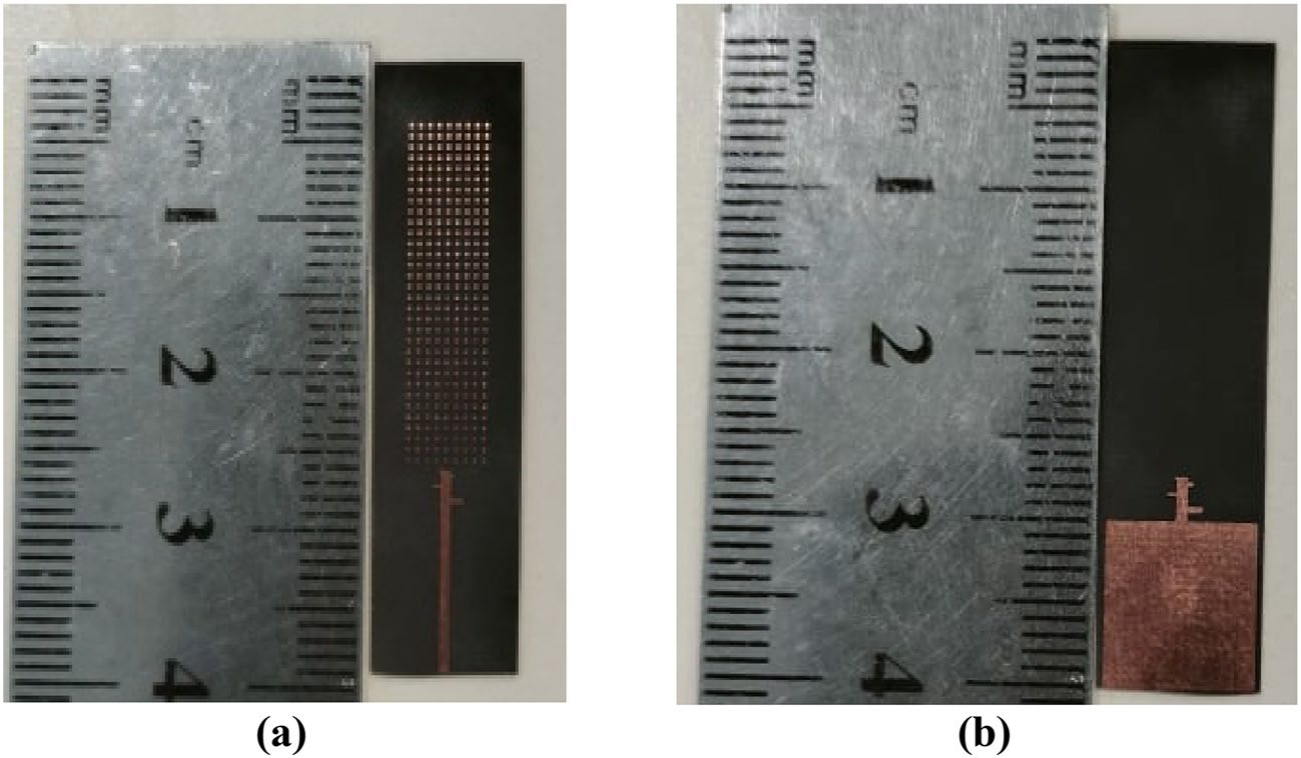
Fabricated loaded LPDA Model, (**a**) Front view, (**b**) Back view.

Due to the insightful analysis, the fabricated, engineered prototype S_11_ was measured by a vector network analyzer with a 50-Ω terminal impedance. The measured results are adjusted for the 50-Ω feeding line losses. The results of the simulated and measured S-parameters are shown in Fig. 11. The measuring antenna reflection coefficient S_11_ is in contrast to the frequency in the assigned super wide band. The S_11_ antenna parameters are evaluated through contrasts between the simulated and physical measurements. The LPDA antenna prototype was fabricated, as is obvious in Fig. 12. The fabrication measurements show that the antenna performs admirably with an S-parameter below − 10 dB over a wide frequency range between 40 GHz and much more than 70 GHz. It is clear that the design has an observed S_11_ of roughly − 34.88 dB and − 20 dB from simulation, as shown in Fig. 11. The measured S_11_ exhibits noise and fluctuation. This noise is due to fixing the connector to the substrate in a complex way due to the small size of the antenna. The connector type is an adjustable Edge Launch Connector, so two holes are needed to fix it to the substrate. The inconsistency observed in the measured results is due to fabrication accuracy and the frequency-dependent permittivity behavior of the substrate at higher frequencies. The measurement equipment's accuracy degradation is a result of the measuring instruments declining precision over time.

**Figure 13:**
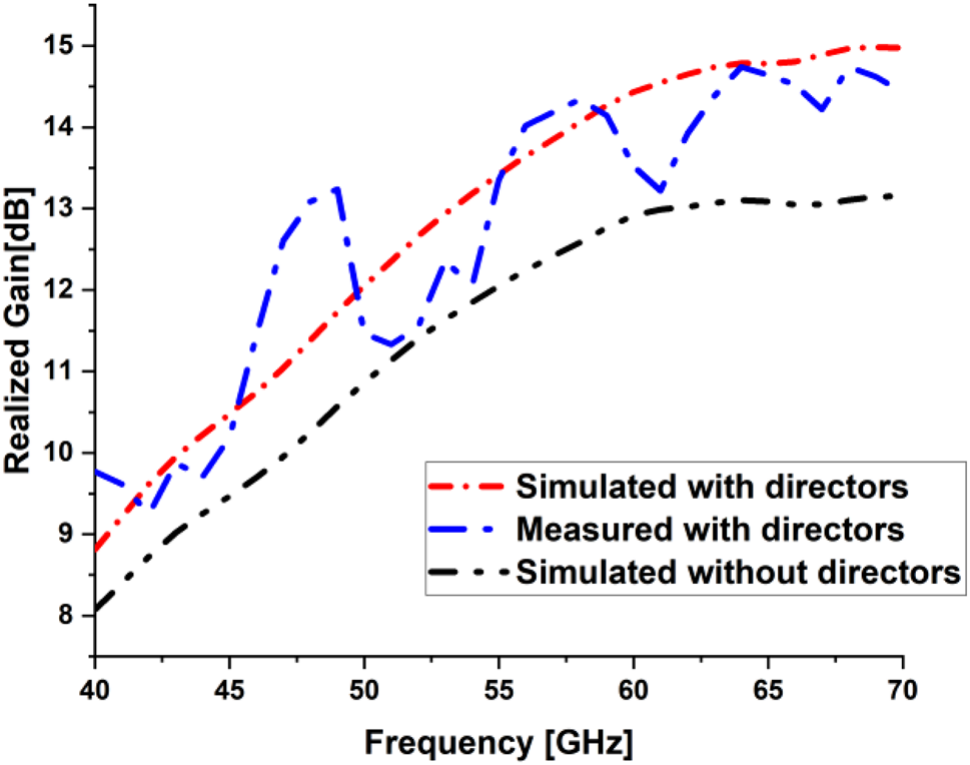
Measured and Simulated PDA Total Realized gain.

**Figure 14:**
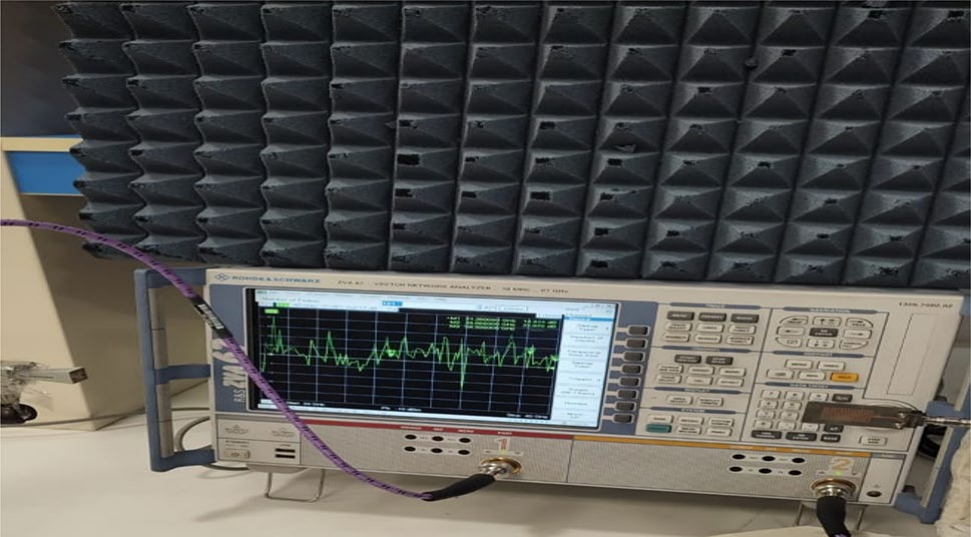
Radiation pattern measurements for the proposed LPDA.

**Figure 15:**
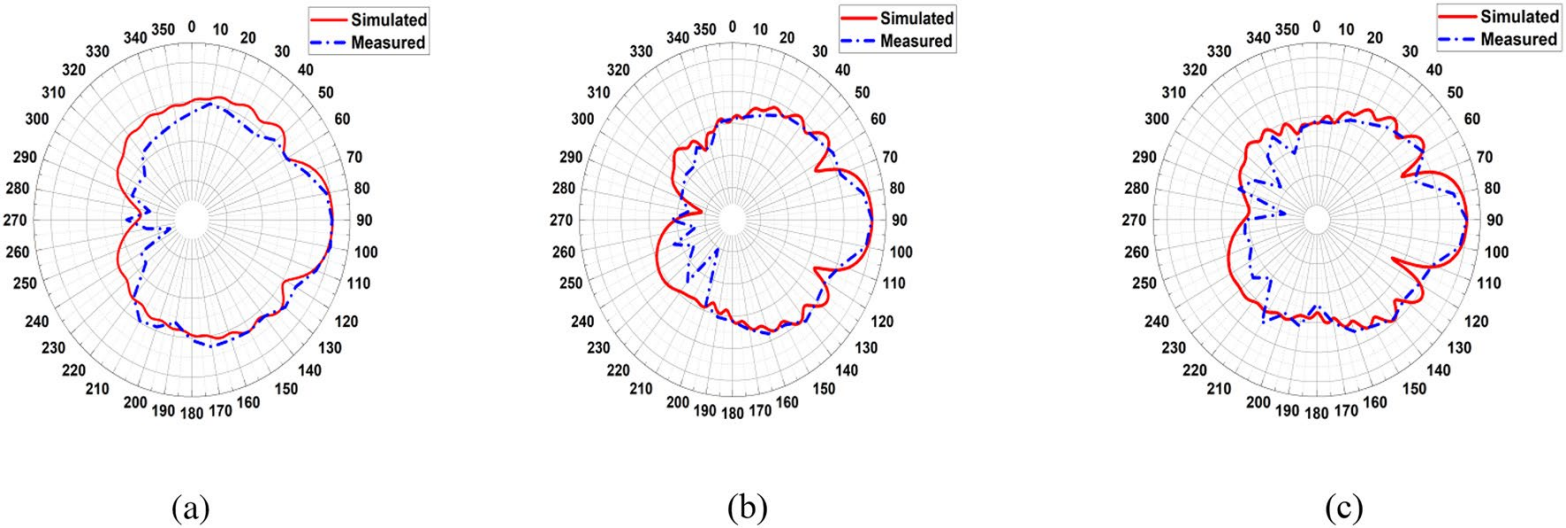
Simulated and Measured Radiation Pattern E-plane: (**a**) 45GHz, (**b**) 55GHz, (**c**) 60GHz.

**Figure 16:**
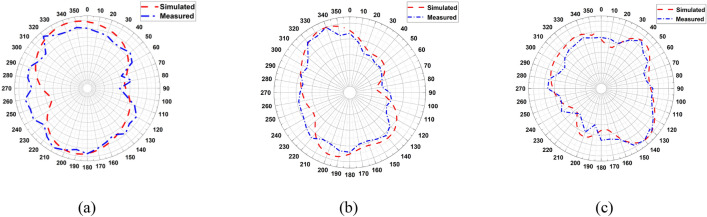
Simulated and Measured Radiation Pattern H-plane: (**a**) 45GHz, (**b**) 55GHz, (**c**) 60GHz.

**Figure 17:**
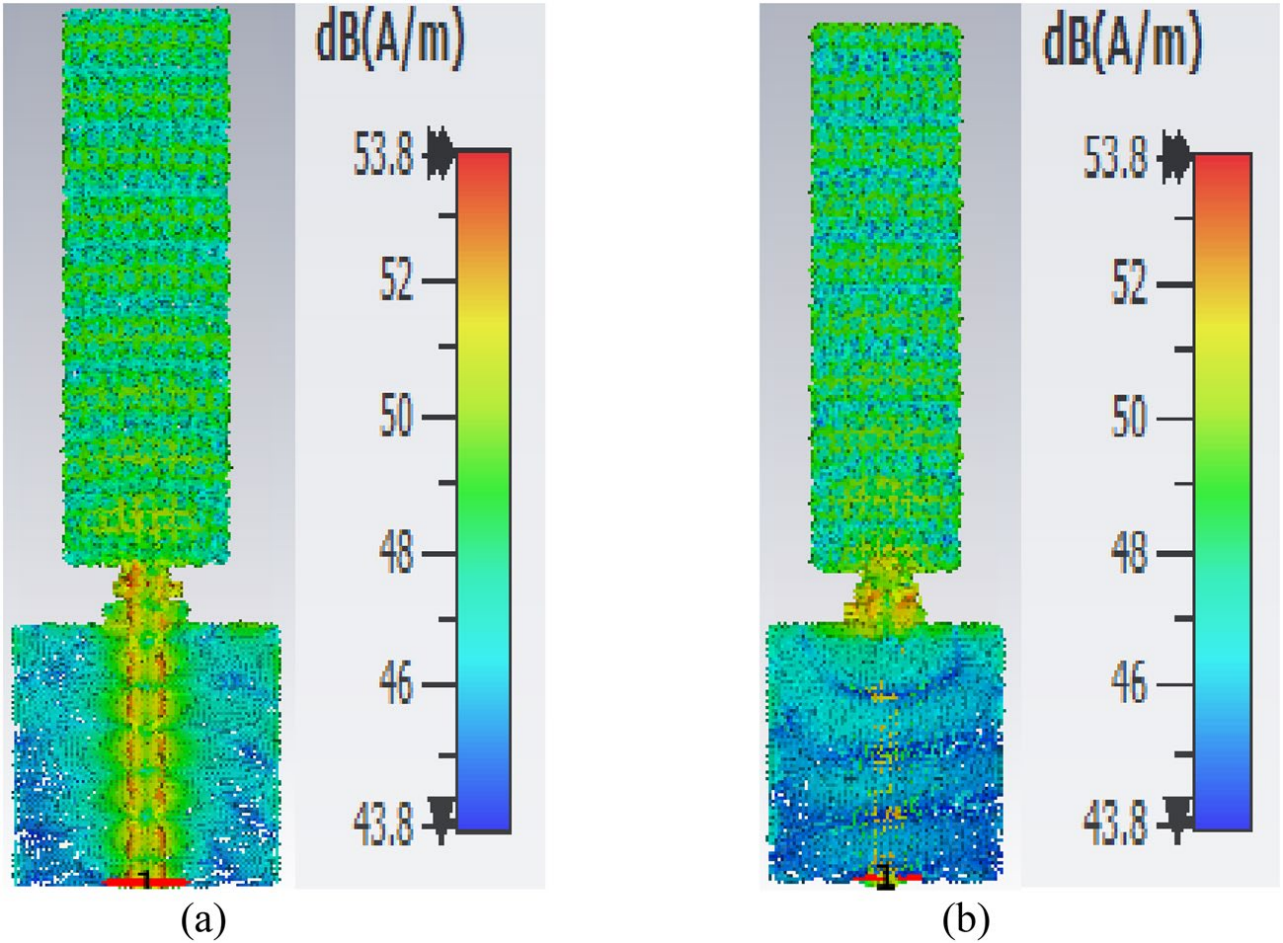
Surface current distributions at the resononat frequency of 60GHz, (**a**) Front View, (**b**) Bck View.

The realized gain of LPDA was simulated and measured. The gain was measured using two sequential and identical horn antennas arranged in a coordinating configuration in line-of-sight. The first is for sending, and the second is for receiving. The Standard Gain Horn Antenna LB-19-20-C-1.85F has a low VSWR of 1.25:1 and an output of 1.85mm with a female connector. It operates between 40 and 60 GHz with a nominal gain of 20 dBi. The LB-19-20-C-1.85F model offers efficient performance characteristics and directionality due to its uniform gain over its frequency span. It can manage 10W of peak power and 5W of continuous power. Linearly polarized, this standard gain-horn antenna is perfect for measuring antenna gain and pattern. The gain increased with the loaded LPDA compared to the unloaded ones, as listed in Table 3. It is demonstrated that the proposed LPDA is superior to the previous work since the gain has been considerably improved, as indicated in Table 4. The frequency range and bandwidth have been expanded. The realized gain for unloaded LPDA has reached 12.78 dBi over 60 GHz, 12.01 dBi over 55 GHz, and 9.308 dBi at 45 GHz. There is a slight difference between the measured and simulated results due to what was precisely measured and what was modeled, which is a result of the measuring instruments declining precision over time, the frequency-dependent measurement equipment's accuracy degradation, reflection, and various regions of the antenna's different edge impedance. It is clear that there has been an overall 3 dBi rise in LPDA gain. The antenna's overall actual gain is 14.97 GHz over 60 GHz, 13.42 dBi over 55 GHz, and 10.48 dBi over 45 GHz, as shown in Table 3. The measured gain for the LPDA was practically measured and tested in Fig. 13. The results are listed in Table 3. To generate 3D radiation characteristics, the tested LPDA antenna is placed on a rotating rod and twisted from 0 to 360 degrees. The measurements of the antenna's two main axes are commonly made to find out specifics, like the beam width in the E and H planes in Fig. 14. In order to determine the radiation pattern, multiple data points need to be collected every 5°. The actual and modeled radiation patterns of the antenna in the E- and H-plane at 45 GHz, 55 GHz, and 60 GHz can be seen in Figs. 15 and 16. The antenna generates a uniform radiation pattern in the E-plane and H-plane and exhibits superior back-lobe suppression. The current distribution on the antenna structure's surface was sufficient to provide further details about the intended performance of the antenna in the frequency range. The surface currents are concentrated through the director radiating elements, so the effect of the constructive coupling on the overall performance of the antenna is this strong coupling causes a lot of energy to be connected to various elements, which causes surface current distributions at 60 GHz. Finally, the majority of the surface current is concentrated at the border of the feed line, nearly attaining a 60 GHz surface current. The surface current is the real current induced by an electromagnetic field and determines the radiation pattern. EM fluctuations create a field in the antenna. The field causes a free electron to move, creating a flow of electric current. The current distributed through the director units at an operating frequency of 60 GHz, as seen in Fig. 17, absorbs a great amount of wave to re-radiate the wave as well, which enhances the gain and radiation pattern. By creating a director's impact, the directors are designed to replace dielectric lenses and metamaterials in order to maximize gain. Metamaterials stimulate novel perspectives on traditional electromagnetic concepts. The metamaterial structures' resonant characteristics and some of the resulting bandwidth limitations are the main drawbacks. Metamaterial-inspired antennas frequently have low gain. In comparison with reflector antennas, these antennas are more expensive for the same gain and range. The antenna impedance matching is enhanced by the directors, as the directors' coupling affects the reflection at the input. The director's spacing becomes a significant factor. Even a little distance between directors significantly reduces the spectrum and gain. If the directors are too distant from one another, there will be significant losses. Directors will increase the gain of the antenna. By reradiating the radio waves with a new phase, they change the radiation patterns of the waves. The end outcome is constructive interference, which reduces backlobe and increases gain by strengthening the overall signal. As declared in the circular, directors support the LPDA arrays. It is possible to achieve the same level of radiation effectiveness because the current generated on the arms is better suited for the propagation of waves.

**Table 3:** Measured and simulated gain.

Frequencies [GHz]	Simulated gain without directors [dBi]	Simulated gain with directors [dBi]	Measured gain with directors [dBi]
45	9.0213	10.48	10.196096
55	12.01	13.42	13.351997
60	12.78	14.97	13.518880

**Table 4:** The proposed work Vs. the previous work.

Reference no	Frequency range [GHz]	Material	Material thickness	Peak gain [dBi]	Bandwidth [GHz]	Impedance bandwidth (%)	Antenna size[mm]	Complexity
^24^	23.41–33.92	RT5880	0.254	10.7	10.51	35.5	5 × 9	Complex
^25^	28–38-55	RT5870	0.79	7.35	15	30	8 × 8	Complex
^26^	25–45	Rogers3003^R^	0.75	10	20	57	150 × 75	Simple
^27^	21–37	RT5880	0.787	12–14.5	16	55	40 × 13	Complex
^28^	26–39	RO5880	0.254	11	13	46	25 × 10	Complex
^29^	23.5–29.4	RT5880	0.254	10.44	5.9	22	24 × 24	Simple
^30^	76/93.7	Roger	0.127	10.90	1.4	22	9 × 8	Moderate
^31^	24–39	RO588	0.508	10.5	15	47	40 × 15	Simple
^32^	34–40	RT-5880	0.254	12.8	6	16	20 × 40	Complex
^33^	26.5–30.4	RT5880	0.508	11	3.9	13	28.3 × 28.3	Complex
^34^	33–40	Rogers 5880	0.787	12	7	20	33.31 × 54.96	Moderate
^39^	1.65–70	RO 3003	1.52	12.89	38.35	52	60 × 40	Complex
^40^	57–84	RO3003	1.52	7	27	38	–	Complex
^41^	5.45–80	Rogers 5880	0.8	8	74.55	57	18 × 12	Simple
^42^	2.23–100	RT /Duroid5880	1.57	9.52	97.77	52	26 × 50	Complex
^43^	12–22	RT-5880	1.57	10.7	10	57	55 × 45	Complex
^44^	25–35.5	Arlon Di clad 880TM	0.02	11.5	10.5	34.73	20 × 38	Complex
^45^	23–40	Rogers-5880	1.57	11.45	17	53	40 × 40	Complex
^46^	2–115	FR-4	1.6	7.23	113	51	16 × 22	complex
Proposed work	40–70	RT5880	0.254	14.97	30	58	39 × 10	Moderate

## Conclusion

In this paper, an operational high-gain and wide-band millimeter-wave LPDA antenna is designed and implemented. The LPDA presents a super-wide band from 40 GHz to well over 70 GHz. The prototype is discussed in detail. It is based on the design and implementation of loaded and unloaded LPDA to enhance the gain, widen the antenna spectrum, reduce the minor lobes, and increase the antenna performance. As a result, this arrangement is created, and its radiation and gain properties are observed through experimentation. This results in an improvement of the suggested antenna's radiation properties within the operating bandwidths. The realized gain for the unloaded LPDA array is 9.0213 dBi, 12.01 dBi, and 12.78 dBi at 45 GHz, 55 GHz, and 60 GHz, respectively. The LPDA with loaded directors produced a peak gain of 14.97 dBi at 60 GHz, 13.42 dBi over 55 GHz, and 10.48 dBi over 45 GHz. The impedance matching of the antenna is 58%. The antenna's radiation characteristics were measured and tested. Several advantages, including a better antenna gain and a steady radiation characteristic, are displayed. The design presents exceptional qualities in the literature that surpass the capabilities of the current antenna based on modeling and experimental data. The antenna is a proper candidate to be used in many V-band applications, like wireless personal area network (WPAN) applications recommended by IEEE 802.11ad and IEEE 802.15.3c.
